# Pharmacodynamics and Pharmacokinetics of HSK3486, a Novel 2,6-Disubstituted Phenol Derivative as a General Anesthetic

**DOI:** 10.3389/fphar.2022.830791

**Published:** 2022-02-03

**Authors:** Juan Liao, Meiting Li, Chaoli Huang, Yan Yu, Yashu Chen, Jiaqi Gan, Jie Xiao, Guilin Xiang, Xizhi Ding, Rong Jiang, Peng Li, Mengchang Yang

**Affiliations:** ^1^ Department of Stomatology, Sichuan Provincial People’s Hospital, University of Electronic Science and Technology of China, Chengdu, China; ^2^ Department of Anesthesiology, Sichuan Provincial People’s Hospital, University of Electronic Science and Technology of China, Chengdu, China; ^3^ East Hospital, Sichuan Provincial People’s Hospital, University of Electronic Science and Technology of China, Chengdu, China; ^4^ Haisco Pharmaceutical Group Co. Ltd., Chengdu, China

**Keywords:** anesthesia, GABA, sedation, pharmacokinetics, pharmacodynamics

## Abstract

**Background:** The purpose of this study was to characterize the novel sedative/hypnotic agent HSK3486, a 2,6-disubstituted alkylphenol analogue.

**Methods:** The mechanism of action of HSK3486 was studied in competitive binding assays and whole-cell patch clamp assays. HSK3486 was administered by bolus intravenous injection to dogs and rats, and the loss of righting reflex as well as effects on the cardiovascular and respiratory systems were assessed. The *in vitro* metabolism of HSK3486 was analyzed by CYP450 genotyping and enzyme inhibition.

**Results:** HSK3486 competed with t-butylbicycloorthobenzoate (TBOB) and t-butylbicyclophosphorothionate (TBPS) for binding to the gamma-aminobutyric acid type A (GABA_A_) receptor. HSK3486 potentiated GABA-evoked chloride currents at lower concentrations while activating GABA_A_ receptor at higher concentrations. HSK3486 induced hypnosis in rats and dogs, and had a higher therapeutic index than propofol in rats. The hypnotic potency of HSK3486 was approximately 4-5 fold higher than that of propofol. HSK3486 exerted minimal effects on the cardiovascular system.

**Conclusions:** HSK3486 is a positive allosteric regulator and direct agonist of GABA_A_ receptor. It has a promising sedative/hypnotic effect and good *in vivo* pharmacokinetic properties, which justify further studies towards its clinical application.

## Introduction

Propofol is the most widely used sedative/hypnotic agent for the induction and maintenance of general anesthesia and sedation of patients in intensive care units (ICUs) (Grasshoff and Antkowiak, 2004; Cravero et al., 2009). Since the late 1980s, propofol has been widely used due to its fast onset and clearance, allowing rapid patient recovery (Walsh, 2018). However, propofol presents some limitations, including pain at the injection site, blood pressure decrease, respiratory depression causing apnea, and infusion syndrome (Rochette et al., 2008; Qiu et al., 2016). Injection pain may depend on the propofol concentration in the injectable emulsion, and infusion syndrome may be caused by a high concentration of lipids in the emulsion (Desousa, 2016).

The 2,6-disubstituted alkylphenol HSK3486 is a novel propofol analogue formulated in an injectable emulsion of medium- and long-chain triglycerides similar to that used for propofol (James and Glen, 1980). HSK3486, like propofol, exerts its sedative and hypnotic effects through gamma-aminobutyric acid type A (GABA_A_) receptors. HSK3486 shows higher liposolubility and potency than propofol. Therefore, for the same level of anesthesia, fewer lipids from the HSK3486 emulsion reach the circulatory system than in the case of a propofol emulsion (Qin et al., 2017).

This study characterized the mechanism of HSK3486 interaction with GABA_A_ receptors *in vitro*, and examined the hypnotic profile of the compound *in vivo*. The pharmacokinetic characteristics of HSK3486 were also evaluated *in vitro* and *in vivo*. The sedative/hypnotic profile after intravenous bolus of HSK3486 was evaluated in rats and dogs, and the effects on the respiratory and cardiovascular systems were assessed using telemetry.

## Materials and Methods

### Animals

All animal studies were conducted in accordance with the Guide for the Care and Use of Laboratory Animals (Eighth Edition) from the Chinese National Research Council and the current United States Department of Agriculture Animal Welfare Act (Anderson et al., 1974; International C, 2011).

The anesthetic effects of HSK3486 were evaluated in Sprague-Dawley rats and the hemodynamic effects of HSK3486 were evaluated in beagle dogs purchased from Charles River (Beijing, China), Covance Research Products (New Jersey, United States) or Beijing Marshall Biotechnology (Beijing, China). For the *in vivo* pharmacokinetic study, Sprague-Dawley rats were obtained from Charles River and beagle dogs from MaxMak Bio (Beijing, China). Tissue distribution and excretion studies were performed in Sprague-Dawley rats provided by Shanghai SIPPR-BK Laboratory Animals (Shanghai, China). All animals were housed at 20–25°C on a cycle of 12 h of light and 12 h of dark and were given free access to food and water, except when fasting was required.

### Compounds

HSK3486 was synthesized by the Haisco Pharmaceutical Group (Chengdu, China) as previously reported (Qin et al., 2017). HSK3486 lipid emulsion was sterilized and formulated as an injectable emulsion similar to the 1% propofol sold by Fresenius Kabi (Schaumburg, IL, United States). This propofol formulation (10 mg/ml, Fresenius Kalbi) was purchased as 20-ml, single-use glass ampoules from Juhui Chemical Technology (Chengdu, China) and used for *in vitro* studies.

### Binding Assays

In order to investigate the potential targets of HSK3486, radioligand binding assays were performed at Eurofins Panlabs (Taiwan, China). A total of 116 tests were performed by standard methods to evaluate binding potency of HSK3486 toward targets. In each test, radioligand was applied in the presence of 1 × 10^−5^ M HSK3486 and free ligand was assayed in order to calculate the rate of competitive binding inhibition.

### GABA_A_ Receptor Functional Assays

The effects of HSK3486 or propofol on GABA_A_ (α_1_β_2_γ_2_) receptor were evaluated in HEK-293 cells stably expressing the receptor using the manual whole-cell patch clamp technique (Chandramani Saxena, 2000; Li et al., 2013). Cells were cultured in Dulbecco’s Modified Eagle Medium (DMEM) supplemented with 10% fetal bovine serum (FBS), 800 μg/ml G418, 200 μg/ml hygromycin B, and 100 μg/ml zeocin. Before experiments, cells were detached using 0.25% trypsin-EDTA. Then 8 × 10^3^ cells were seeded into 24-well plates with cover slips in 500 μL of medium, and experiments were performed 18 h later. The currents were recorded using a gap-free mode at a holding potential of −70 mV. Each concentration of HSK3486 or propofol was applied at least three times, followed by a 1-min wash in wash buffer [5 mM HEPES (pH 7.4), 140 mM NaCl, 5 mM CsCl, 2 mM CaCl_2_•2H_2_O, 1 mM MgCl_2_•6H_2_O, 10 mM D-glucose]. Data were collected with an EPC-10 amplifier and stored on a computer using PatchMaster software (both from HEKA Elektronik, Lambrecht, Germany), then analyzed using Graphpad 8.0 software (Prism, San Diego, CA, United States).

In potentiator experiments, HSK3486 or propofol was applied for a 45-s pre-incubation followed by a 2-s co-application with 3 μM GABA. In agonist experiments, the concentration-response curve of HSK3486 or propofol was obtained by exposing cells to increasing concentrations of HSK3486 or propofol alone, with each concentration applied 3–4 times. The current responses to HSK3486 or propofol were normalized to a positive control (300 µM GABA). Means and standard errors were calculated for each test group. Curve-fitting and EC_50_ calculations were performed using Graphpad 8.0.

### Anesthetic and Hemodynamic Effects of HSK3486 in Rats

A rat model was used to evaluate the anesthetic potency of HSK3486. Sprague-Dawley rats were split into 20 groups of 10 animals each (five male, five female). Rats in each group were given vehicle, HSK3486 or propofol by intravenous (IV) administration in the lateral tail vein during 10 s. A narrow set of doses was chosen based on whether it triggered loss of righting reflex (LORR) (Mccarren et al., 2013). The anesthetic effect of HSK3486 was assessed as previously described (Qin et al., 2017). The onset and duration of hypnosis as well as the recovery profile following return of consciousness were recorded (Qin et al., 2017).

Hemodynamic parameters in treated animals were measured using telemetry (Data Sciences International, New Brighton, MN, United States). Briefly, eight male rats carrying a telemetry transmitter received escalating IV bolus doses of HSK3486 or propofol. A balanced latin-square crossover design was applied for dose assignments (D'Enes et al., 1992). HSK3486 was tested at 2 and 4 mg/kg, while propofol was tested at 8 and 16 mg/kg (*n* = 6–8 animals per dose). Each animal received the injection 3–4 times, and the wash-out period between injections was at least one day. Mean arterial blood pressure (MAP), heart rate (HR), and body temperature were recorded every minute from before dosing to 30 min after dosing. All animals recovered righting reflex within 12 min.

### Anesthetic, Cardiovascular, and Respiratory Effects of HSK3486 in Beagle Dogs

The anesthetic effect was evaluated in beagle dogs based on the loss of eyelid reflex and leg withdrawal reflex following a toe pinch every 15 s. Six beagle dogs (three male, three female) weighing 8–10 kg received a single bolus IV injection of HSK3486 or propofol at a range of concentrations. A double latin-square design was applied. The administration interval was 2 days. The anesthetic effect of HSK3486 was assessed using: 1) onset time, defined as the interval from administration until loss of eyelid reflex or leg withdrawal reflex; 2) duration time, defined as the interval from onset of anesthesia until recovery of eyelid or leg withdrawal reflex; and 3) recovery time, defined as the interval from recovery of anesthesia until normal ambulation.

In separate experiments, beagle dogs were also used to evaluate the cardiovascular and respiratory effects of HSK3486-induced anesthesia. These experiments were performed at Joinn Laboratories (Suzhou, China). Eight beagle dogs (four male, four female) weighing 7–10 kg were quarantined, acclimatized, implanted with telemetry transmitters, and allowed to recover from the implantation surgery. A double latin-square design was applied. HSK3486 was given by IV administration at dosages of 1, 2, and 4 mg/kg; the injection was complete within 60 s. The administration interval was 2 days. Dogs were monitored for 3–5.5 h before administration and in the intervals between data collection timepoints. The clinical observations were performed twice daily (am and pm) on the other days. MAP, electrocardiography, body temperature, HR, and respiratory functions were assessed for 8 h after dosing; these data were acquired and analyzed using the Ponemah Physiology Platform 4.8 (Valley View, Ohio, United States).

### Single-Dose Pharmacokinetics of HSK3486 in Rats

Equal numbers of male and female Sprague-Dawley rats were allocated into two groups of six animals each and given HSK3486 at1,2 or 4 mg/kg dose in order to mirror the hemodynamic study. Blood from each animal was sampled into heparin-containing tubes before dosing and at 2, 4, 8, 12, 20, 30, 60, 90, 120, and 180 min afterward. Samples were placed on ice until centrifugation, after which plasma supernatant was transferred to a clean tube and stored at −70°C until analysis by liquid chromatography-tandem mass spectrometry (LC-MS/MS).

### Single-Dose Pharmacokinetics of HSK3486 in Beagle Dogs

Equal numbers of male and female beagle dogs were divided into three groups of six animals each. Animals were fasted (with free access to water) for 12 h before dosing. Dogs in each group received HSK3486 at 1, 2, or 4 mg/kg in an injection volume of 0.5 ml/kg. Each injection lasted approximately 15 s. Blood was sampled and analyzed as described above.

### Binding of HSK3486 to Dog, Rat or Human Plasma Proteins *in vitro*

HSK3486 working solution (50.0 μg/ml) was diluted with dog, rat or human plasma to a final concentration of 80, 400, or 1,200 ng/ml. Samples of each concentration for every species were obtained immediately or after incubation, and then centrifuged for 16 h at 4°C to obtain supernatants, which were stored at −70°C. Total drug concentration (C_0_) was calculated by analyzing supernatants before storage, while free drug concentration (C_f_) was calculated by analyzing supernatants after 4 h incubation at 4°C. Plasma protein binding rate (%) was calculated as (C_0_-C_f_)/C_0_ × 100 (Han et al., 2013).

### Tissue Distribution of HSK3486 After IV Administration in Rats

Equal numbers of male and female Sprague-Dawley rats were allocated to groups of six animals each and given a bolus injection of 2 mg/kg HSK3486 *via* the tail vein. Rats in each group were sacrificed at 4, 20, 60, or 240 min after administration, and the following tissues were sampled: whole blood, brain, heart, liver, lung, kidney, bladder, pancreas, testis, ovary, uterus, stomach, duodenum, adrenal gland, skin (abdomen), skeletal muscle, and fat. Plasma was prepared from collected whole blood. All samples were stored at approximately −70°C until analysis by liquid chromatography (Acquity UPLC, Waters, United States) followed by tandem quadrupole time-of-flight mass spectrometry (AB SCIEX, Foster City, CA).

### Ability of HSK3486 to Inhibit CYP450 Isoenzymes in Human Liver Microsomes *in vitro*

The inhibitory effect of HSK3486 on cytochrome P450 superfamily members CYP1A2, CYP2B6, CYP2C8, CYP2C9, CYP2C19, and CYP2D6 activity was evaluated based on the production of metabolites in an *in vitro* assay involving purified microsomes (Maria et al., 2020).

HSK3486 was dissolved to 100 mM in dimethyl sulfoxide (DMSO). Stock solutions of the following CYP450 substrates were prepared in methanol: phenacetin (50.0 mM), bupropion (50.0 mM), paclitaxel (20.0 mM), tolbutamide (100 mM), S-mephenytoin (100 mM), dextromethorphan hydrobromide (10.0 mM), midazolam (5.00 mM), and testosterone (50.0 mM). All these solutions were diluted with phosphate-buffered saline (PBS) in assays. Reactions were pre-incubated for 5 min at 37°C, then reduced nicotinamide adenine dinucleotide phosphate (NADPH) was added. Reactions were terminated at suitable time points by adding an equal volume of ice-cold acetonitrile, then analyzed for cytochrome P450 metabolites using a validated method based on liquid chromatography-tandem mass spectrometry (Nithipatikom et al., 2001). The half-maximal inhibitory concentration (IC_50_) was calculated for HSK3486 against each cytochrome P450 isozyme.

### *In vitro* Metabolism of HSK3486

Metabolism of HSK3486 was assessed in the presence of microsomes or S9 fraction (Qin et al., 1983) purified from the liver of humans, monkeys, dogs, rats, and mouse. The investigations were carried out under contract at Shanghai Institute of Materia Medica (555 Zu Chong Zhi Road, 211203 Shanghai, PRC). The microsome assay contained 3.0 µM HSK3486, 3.2 mM MgCl_2_, 1.0 mM uridine 5′-diphosphoglucuronic acid (UDPGA), 25.0 μg/ml alamethicin, and microsomes (0.5 mg protein/mL) in 100 mM PBS (pH 7.4). The mixture was pre-incubated for 3 min at 37°C open to the air. Then NADPH was added to initiate the reaction, and the mixture was incubated at 37°C for 60 min. The reaction was terminated by adding an equal volume of ice-cold acetonitrile.

The S9 assay was conducted at 37°C for 1 h in Tris-HCl buffer (100 mM, pH 7.4) containing 5.0 mM MgCl_2_, 3.0 µM HSK3486, 2.0 mM UDPGA, 0.5 mM 3′-phosphoadenosine-5′-phosphosulfate (PAPS), 25.0 μg/ml alamethicin, 8 mM DTT, 0.1% BSA, and liver S9 fraction (2.0 mg protein/mL). The mixture was pre-incubated for 3 min at 37°C open to the air, then 1.0 mM NADPH was added to initiate the reaction, which was incubated at 37°C for 60 min. Two of the triplicate samples were terminated by addition of an equal volume of ice-cold acetonitrile. The third triplicate sample received glucuronidase in citric acid buffer (pH 5.0) and was incubated another 16 h at 37°C. The reaction was then terminated by addition of 200 μL of acetonitrile.

All samples from the microsomes and S9 assays were vortexed, centrifuged for 5 min (14,000 *g*), then analyzed by ultra-performance liquid chromatography (LC-30AD, Shimadzu, Kyoto, JP) followed by quadrupole time-of-flight mass spectrometry (AB Sciex).

### Identification of Cytochrome P450 Enzymes Responsible for Metabolism of HSK3486

Two test systems were used to determine which CYP enzymes are responsible for the metabolism of HSK3486. The investigations were carried out under contract at Pharmaron (6 Taihe Road, 100176 Beijing,PRC). In one test system, HSK3486 and chemical inhibitors were incubated with human liver microsomes (0.5 mg/ml, Corning, New York,United States) in a total volume of 100 μL PBS (100 mM, pH 7.4). After a 3-min pre-incubation in an open water bath at 37°C, the reaction was initiated by addition of 1.0 mM NADPH and allowed to continue for 60 min, then terminated by addition of an equal volume of ice-cold acetonitrile. All reactions were conducted in polypropylene tubes in duplicate.

In another test system, 3.0 µM HSK3486 was incubated with recombinant human P450 isoenzymes at 37°C for 1 h in 100 mM PBS (pH 7.4) containing 2.0 mM NADPH in a water bath open to the air. After the mixture of HSK3486 and NADPH had equilibrated for 3 min at 37°C, 50 pmol/ml of one of the following recombinant human P450 isoenzymes was added: 1A2, 2A6, 2B6, 2C8, 2C9, 2C19, 2D6, 2E1, 3A4, or 3A5. Reactions were terminated by adding an equal volume of ice-cold acetonitrile. Incubations were performed in duplicate.

All samples were mixed by vortexing, then centrifuged at 14,000 *g* for 5 min. The supernatant was transferred to a clean tube and mixed with an equal volume of 0.001% ammonia in 5 mM ammonium acetate. A 10-µL aliquot of the mixture was analyzed by ultra-performance liquid chromatography (LC-30AD, Shimadzu, Kyoto, JP) followed by quadrupole time-of-flight mass spectrometry (AB Sciex, Foster City, CA, United States).

### Statistical Analysis

IC_50_ values in binding assays were determined by non-linear least-squares regression using MathIQTM (ID Business Solutions, United Kingdom). Inhibition constants (K_i_) were calculated from observed IC_50_ values using the equation of Cheng and Prusoff (Cheng, 1973). EC_50_ values in receptor function assays were calculated by non-linear regression of log (concentration)-response curves using graphpad 8.0(Prism, San Diego, CA, United States): Y=Bottom + (Top-Bottom)/(1 + 10^((LogEC50-X)*HillSlope)) (Gadagkar and Call, 2015).

A non-linear fitting method was applied to calculate the hypnotic activity in rats at 10 and 50 min after LORR (HD_10_, HD_50_). A non-compartmental model was applied to calculate the pharmacokinetic parameters using Phoenix WinNonlin 6.3 software (Pharsight, New York, United States). For the metabolism study, data were collected using Analyst^®^TF 1.6 (AB Sciex, Foster City,CA,United States) and Masslynx 4.1 (Waters), and analyzed using PeakView^®^1.2 and MetabolitePilot 1.5 (AB Sciex, Foster City, CA, United States). In assays of HSK3486 inhibition of CYP450 isoenzymes in human liver microsomes, IC_50_ values were calculated by probit analysis using Origin 6.0 and SPSS 17 (Chicago, IL, United States).

## Results

### Binding Assay

Of the 116 potential targets tested, 10 μM HSK3486 produced > 50% inhibition of specific binding only at GABA_A_ chloride channels, norepinephrine transporter, and CPY2C19 (Supplementary Table S1). Among the GABA_A_ receptor ligands muscimol, t-butylbicyclophosphorothionate (TBPS), (Squires and Saederup, 1987; Van Rijn et al., 1990) flunitrazepam, and Ro-15-178, all of which bind at different sites, HSK3486 only affects TBPS binding to GABAa receptor.

### Direct Active and Amplifying Effects of HSK3486 on GABA_A_ Receptor

**FIGURE 1:**
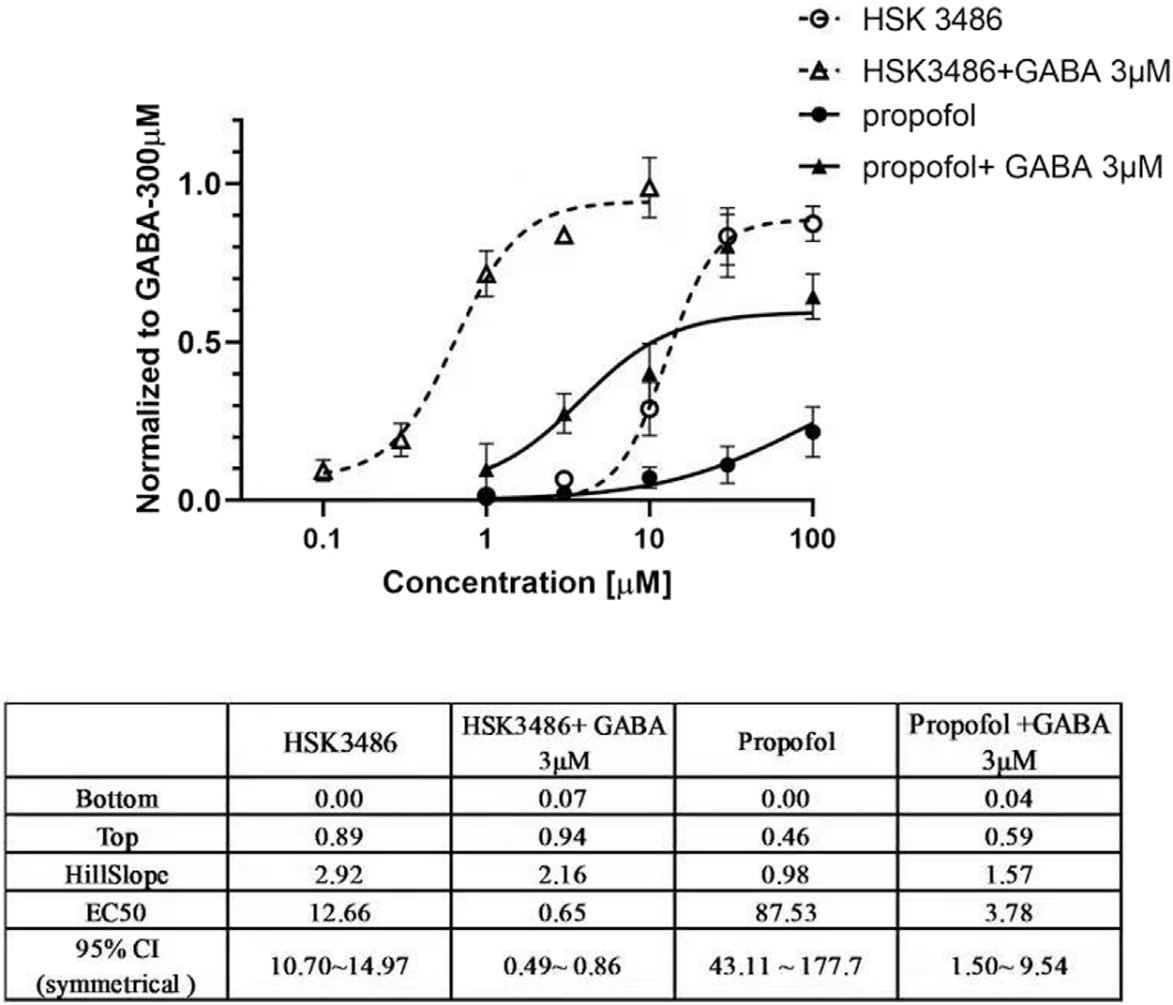
Potentiator and direct agonist effects of HSK3486 on gamma-aminobutyric acid type A (GABA_A_) receptors. Data show the mean current increase ± SD evoked by3 μM GABA in the presence or absence of HSK3486 or propofol, or evoked by each of the respective drugs on its own. The current increase is shown as a fold difference from the current observed with 300 μM GABA.

Both HSK3486 and propofol potentiated chloride currents evoked by 3 μM GABA and also directly evoked chloride currents in HEK293 cells transfected with recombinant α_1_β_2_γ_2_ GABA_A_ receptor. HSK3486 exhibited a significant amplifying activity at much lower concentrations than those required for its direct active effects. The maximal peak current evoked by HSK3486 was approximately 90% of that evoked by 300 μM GABA, much higher than the 40% evoked by propofol (Figure 1).

### Anesthetic Effect of HSK3486 in Rats

**FIGURE 2:**
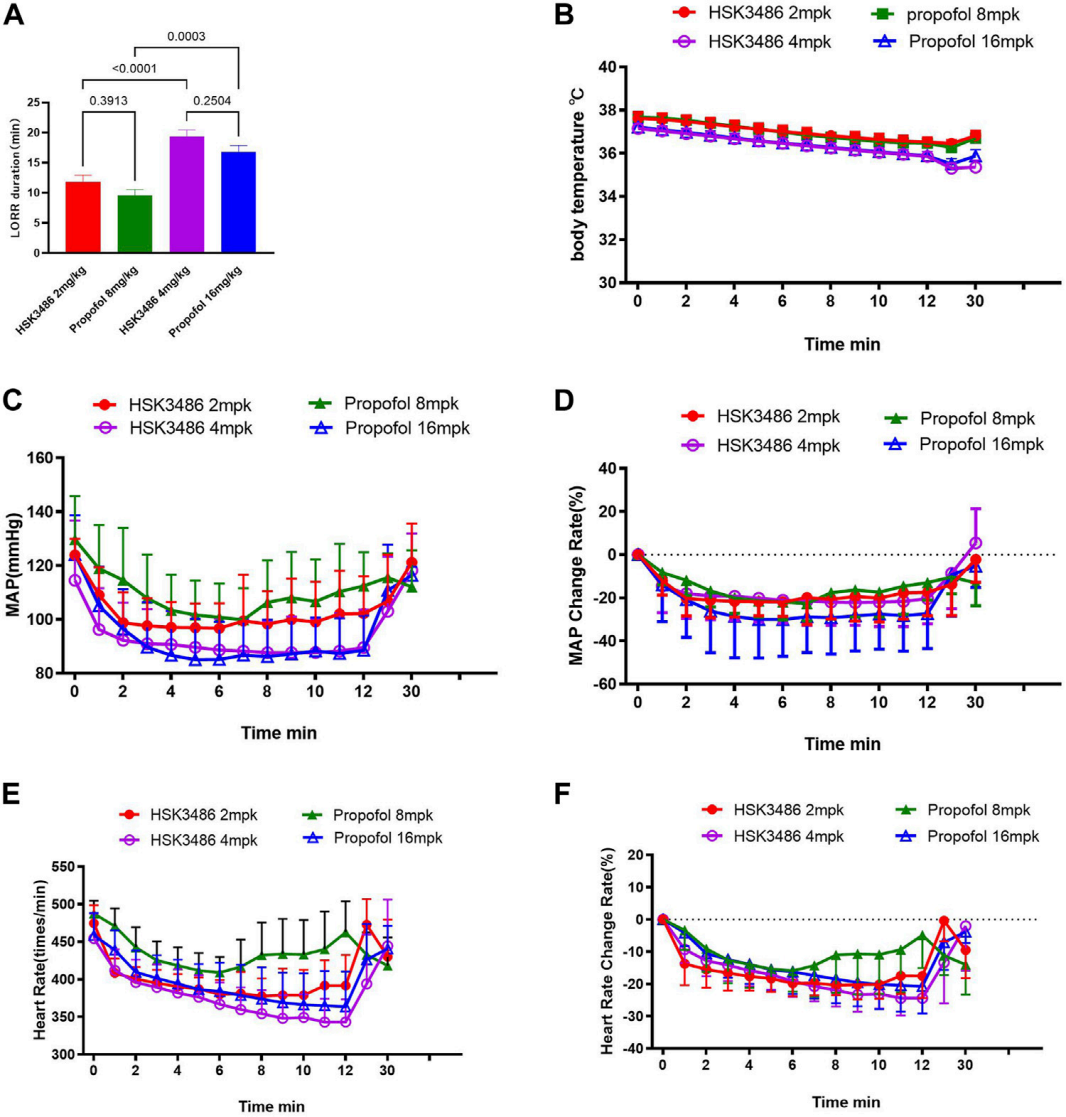
Telemetry measurements in rats after intravenous bolus injection of HSK3486 or propofol. **(A)** Duration of the loss of righting reflex (LORR). **(B)** Body temperature. **(C)** Mean arterial blood pressure (MAP). **(D)** Change in MAP rate. **(E)** Heart rate. **(F)** Change in heart rate. Data are mean ± SD.

HSK3486 showed better anesthesia potency over propofol, with an 83% lower HD_50_. Moreover, HSK3486 showed higher therapeutic index (TI), indicating a better safety window than propofol (Supplementary Table S2). LORR was observed in all animals at HSK3486 doses of 2.0 mg/kg and higher. LORR duration increased with increasing dose (Supplementary Table S3). The animals showed ataxia and weak grip consistent with light sedation from the time when they regained the righting reflex until they could ambulate and grip normally. Sleeping duration with HSK3486 at doses of 2 and 4 mg/kg was similar to that with propofol at respective doses of 8 and 16 mg/kg (Figure 2A).

MAP decreased significantly after injection of HSK3486 or propofol, and recovered to baseline levels within approximately 30 min (Figure 2C). MAP change at high dose was lower in the HSK3486 group than in the propofol group (Figure 2D). HR decreased after administration of HSK3486 or propofol, and it recovered around 7 min later, even returning to baseline levels in groups treated with 4 mg/kg HSK3486 or 16 mg/kg propofol (Figures 2E,F). Body temperature decreased after injection of HSK3486 or propofol, and the magnitude of the decrease was similar between the drug doses that also produced similar anesthetic effect (Figure 2B).

### Single-Dose Pharmacokinetics of HSK3486 in Rats

There were no marked or consistent differences in HSK3486 pharmacokinetics between male and female rats. HSK3486 exposure based on mean C_max_, AUC_0-∞_ and AUC_0-t_ increased in approximate proportion to dose. The average plasma clearance was 15.7 L kg^−1^·h^−1^, and the average Vss was 7.79 L/kg, which was much higher than the total volume of body fluid, indicating a wide distribution of HSK3486. Average plasma elimination half-life was 0.72 h.

**FIGURE 3:**
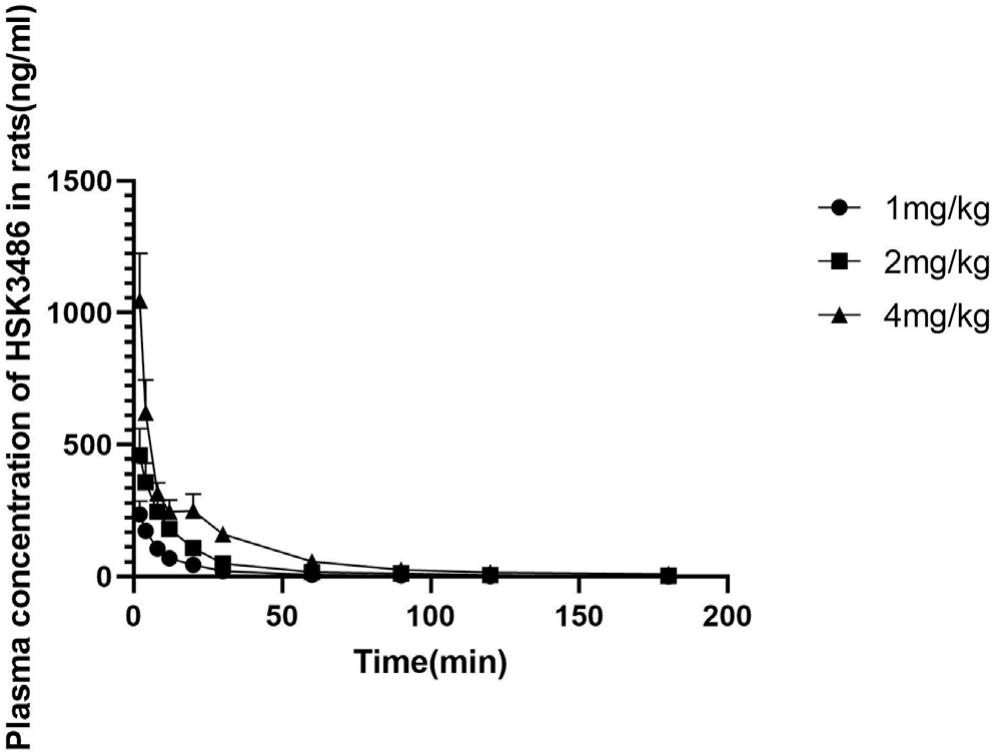
Mean plasma concentration of HSK3486 after intravenous bolus injection in rats. Data are mean ± SD (*n* = 6). Rats in each group received HSK3486 at 1, 2, or 4 mg/kg in an injection volume of 0.5 ml/kg. Blood samples were obtained from each animal at pre-dose and at 2, 4, 8, 12, 20, 30, 60, 90, 120, and 360 min after the dose. Samples were placed in tubes containing K2EDTA and stored on ice until centrifuged to separate plasma. Plasma samples were stored frozen at approximately −70°C until analyzed by a validated method for HSK3486.

HSK3486 mean plasma concentration over time is shown in Figure 3, and pharmacokinetic parameters for HSK3486 in rats are presented in Supplementary Table S4.

### Anesthetic Effect of HSK3486 in Beagle Dogs

HSK3486 or propofol showed a dose-dependent anesthetic effect. The onset time, anesthesia time, and recovery time observed with HSK3486 were similar to those observed with 75% higher doses of propofol. At drug doses associated with the same anesthesia time, animals in the HSK3486 group start of waking at an earlier timepoint after administration (Supplementary Table S5).

### Telemetry Measurement of HSK3486 Effects in Beagle Dogs

**FIGURE 4:**
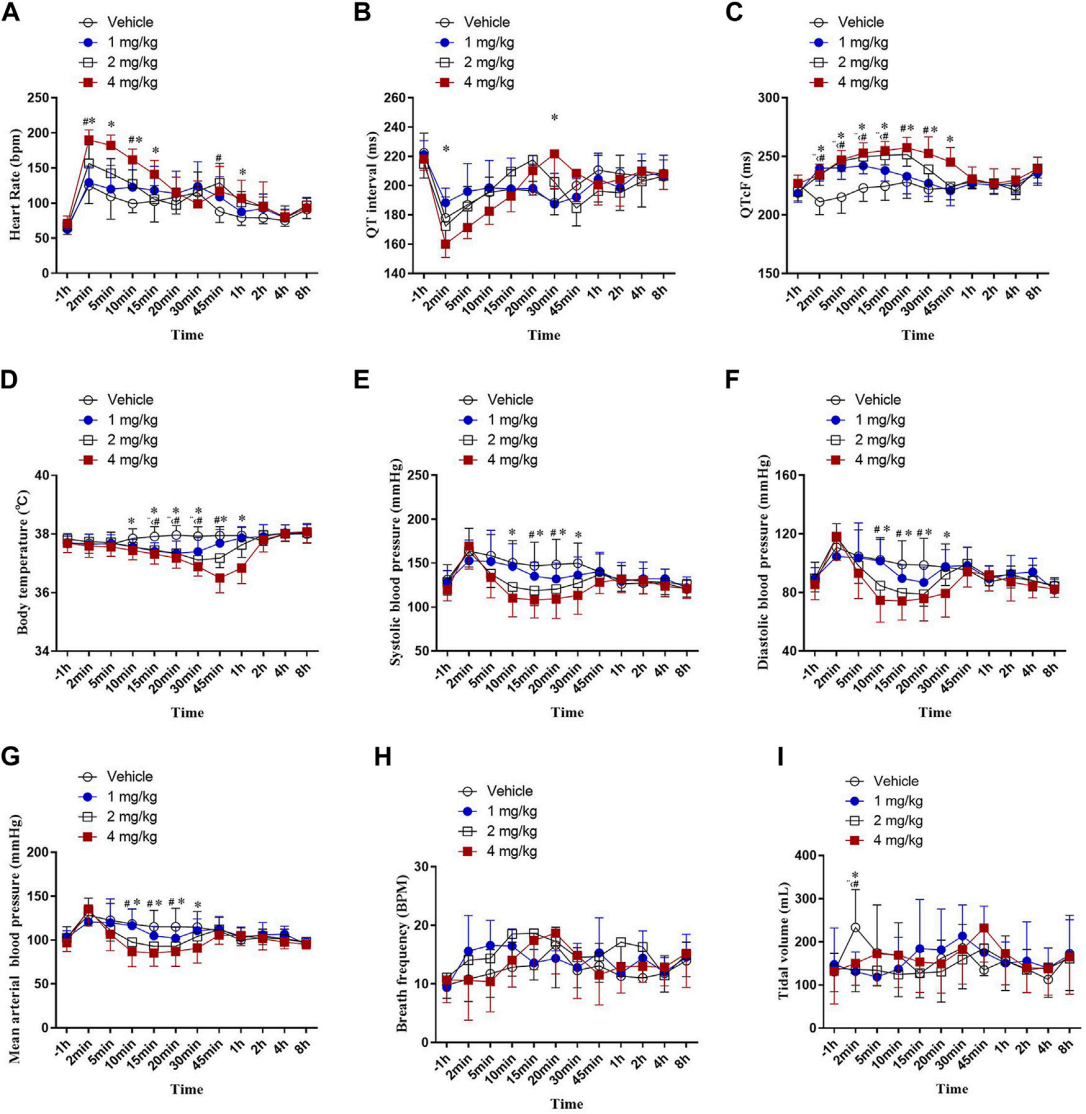
Telemetry measurements after bolus injection of HSK3486 or propofol in beagle dogs (*n* = 8). **(A)** Heart rate. **(B)** QT interval. **(C)** QTc interval. **(D)** Body temperature. **(E)** Systolic blood pressure. **(F)** Diastolic blood pressure. **(G)** Mean arterial pressure. **(H)** Respiratory frequency. **(I)** Tidal volume.

In our experiment, about 2 min after vehicle and ascending dose of HSK3486 administration, a transient tachycardia occurred in all groups. δ% increase of HR were 88, 106, 134 and 169% in vehicle and HSK3486 1,2, 4 mg/kg group respectively. A significant transient increase in heart rate in dogs induced by propofol anesthesia has been reported in many literatures (Pagel and Warltier, 1993; Wouters et al., 1995; Andrea et al., 2018). But in this study, even the injection of vehicle caused a marked increase in heart rate, and there was no significant change in respiratory rate or tidal volume in each group. We hypothesized that in addition to the inhibition of anesthetics on the sympathetic, parasympathetic, cardiac conduction system and working cells of the myocardium, the lack of adequate training in the animals before the experiment, and the stress of the animals after the injection also contributed to the tachycardia. (Figure 4A). After administration of 1, 2, or 4 mg/kg HSK3486, QT intervals were not prolonged (Figure 4B). However, after correction with Fridericia’s formula, the QTcF intervals were significantly prolonged, by up to 17.4%, in a dose-dependent manner within 1 h after HSK3486 administration (Figure 4C). Significant decreases in body temperature were noted within 1 h after administration of 1, 2 or 4 mg/kg HSK3486, with the greatest decrease being 3.85% at 4 mg/kg. Body temperature did not fluctuate substantially during the period from 1 to 8 h after drug administration (Figure 4D).

Blood pressure significantly diminished in a dose-dependent manner within 1 h after administration of 2 or 4 mg/kg HSK3486. Systolic blood pressure decreased by up to 18.8% (Figure 4E); diastolic blood pressure, by up to 23.3% (Figure 4F); and MAP, by up to 21.3% (Figure 4G). Respiratory frequency was not significantly altered after administration of 1, 2, or 4 mg/kg HSK3486 (Figure 4H). All three doses of drug significantly reduced tidal volume at 2 min post-dosing, but not at other time points (Figure 4I).

### Single-Dose Pharmacokinetics of HSK3486 in Beagle Dogs

**FIGURE 5:**
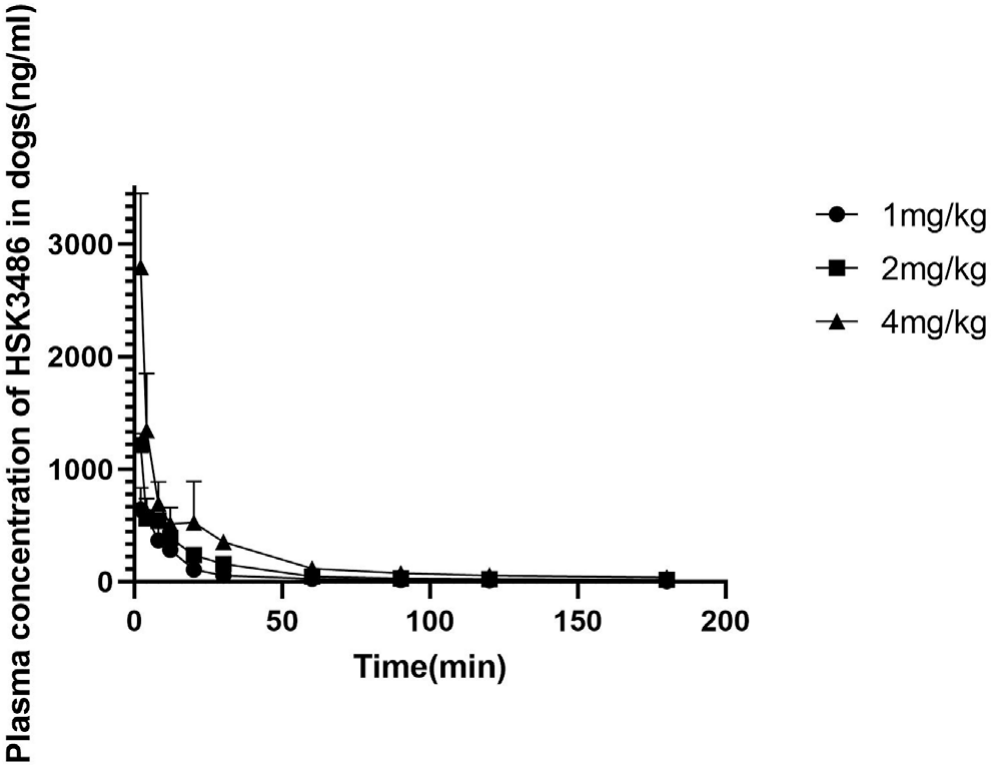
Mean plasma concentration of HSK3486 by time after intravenous bolus injection in beagle dogs. Data are mean ± SD (*n* = 6). Dogs (3/sex/group) were given HSK3486 at doses of 1, 2, or 4 mg/kg by IV injection. Blood samples were obtained from each animal at pre-dose and at 2, 4, 8, 12, 20, 30, 60, 90,120, 240, and 360 min after the dose.

There were no differences in HSK3486 pharmacokinetic parameters between male and female dogs. Exposure based on mean C_max_, AUC_0-A_, and AUC_0-t_ increased with increasing drug dose. The average plasma clearance was 5.75 L kg^−1^·min^−1^, and the average Vss was 6.06 L/kg, which was higher than the total fluid volume in dogs, indicating that HSK3486 was widely distributed. Average plasma elimination half-life was 1.44 h. Mean plasma concentration of HSK3486 with time is shown in Figure 5, and pharmacokinetic parameters for HSK3486 are summarized in Supplementary Table S6.

### Plasma Protein Binding by HSK3486

Binding rates of HSK3486 to human plasma proteins were 96.6 ± 0.4% at 80 ng/ml, 94.6 ± 0.5% at 400 ng/ml, and 93.5 ± 0.3% at 1,200 ng/ml. The corresponding rates of binding to dog plasma proteins were 87.9 ± 0.7%, 91.1 ± 0.2%, and 93.4 ± 0.5%. The corresponding rates of binding to rat plasma proteins were 94.3 ± 0.9%, 94.9 ± 0.2%, and 85.3 ± 0.1%. Thus, HSK3486 bound extensively to plasma proteins from all three species.

### Tissue Distribution of HSK3486 in Rats

**FIGURE 6:**
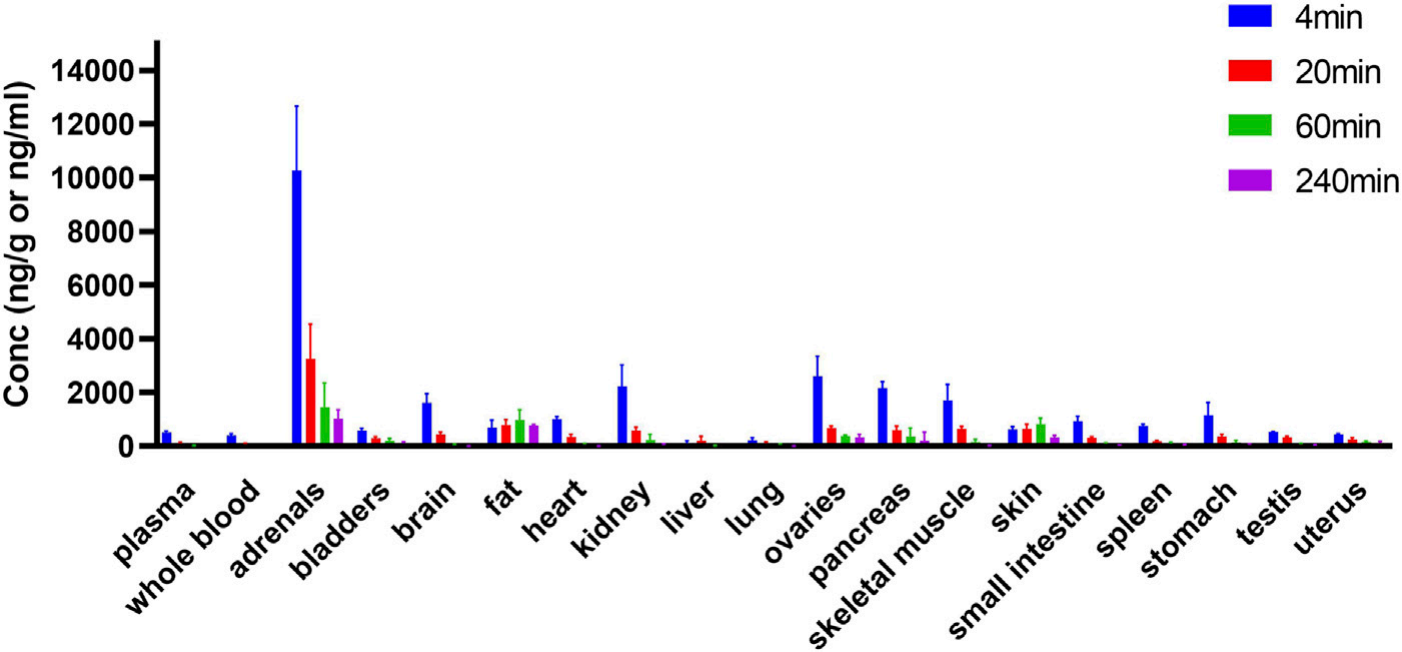
Tissue distribution of HSK3486 in rats following bolus injection (*n* = 6 for each point). Twenty four SD rats were randomly assigned to 4 groups, 3/sex/group and dosed 2 mg/kg HSK3486 by bolus injection. Dosed volume was 5 ml/kg. 4, 20, 60 and 240 min after administration, rats were sacrificed and whole blood, as well as brain, heart, liver, lung, kidney, bladder, pancreas, testis, ovarian, uterus, stomach, duodenum, adrenal gland, skin (belly), skeleton muscle and fat were collected. Plasma was prepared from collected whole blood. HSK3486 concentration were quantitatively determined by validated LC/MS/MS method.

HSK3486 was widely distributed in rats after injection, and it was present mostly in adrenal glands, fat, skin, ovary, pancreas, and kidney (Figure 6). Concentrations of HSK3486 were more than five-fold higher in these tissues than in plasma. Concentrations of HSK3486 were 3.2-fold higher in the brain than in plasma, indicating that HSK3486 can cross the blood-brain barrier. In most tissues, the time to reach the peak concentration was the same as that in plasma (4 min). HSK3486 was rapidly eliminated from most tissues, with less than 10% of peak concentration left at 240 min post-administration, except in fat, skin, bladder, and uterus. The ratio of HSK3486 concentration in whole blood to that in plasma was 0.78, demonstrating low distribution of HSK3486 to red blood cells.

### HSK3486 Inhibition of CYP450 Isoenzymes in Human Liver Microsomes *in vitro*

HSK3486 at 10.0 µM allowed residual activity of 87.0% for CYP1A2, 76.0% for CYP2C8, 63.7% for CYP2D6, and 73.4% for CYP3A4 relative to the activity observed after treatment with midazolam. In fact, IC_50_ values could not be determined for the four isozymes even at HSK3486 concentrations >100 μM. HSK3486 at 10.0 µM allowed residual activity of 52.4% for CYP2B6, 60.5% for CYP2C9, 62.4% for CYP2C19, and 60.4% for CYP3A4 relative to the activity observed with testosterone. IC_50_ values (μM) were 1.53 for CYP2B6, 43.5 for CYP2C9, 5.54 for CYP2C19, and 48.9 for CYP3A4.

### Metabolism of HSK3486 *in vitro*

HSK3486 was not metabolically stable in a mixed UDPGA and NADPH incubation system or in liver S9 fractions from five animal species. HSK3486 was not detected after 60-min incubation; instead, metabolites were detected in microsome extracts from various species: humans, 6 metabolites; monkey, 10 metabolites; dog, 5 metabolites; rat, 5 metabolites; and mouse, 6 metabolites. The major metabolites in human liver microsomes were the hydroxylated metabolites M7-1 and M7-2. The major metabolites in human liver S9 fractions were glucuronide conjugates of the parent drug (M4) as well as M7-2 (M5-1 and M5-2).

**FIGURE 7:**
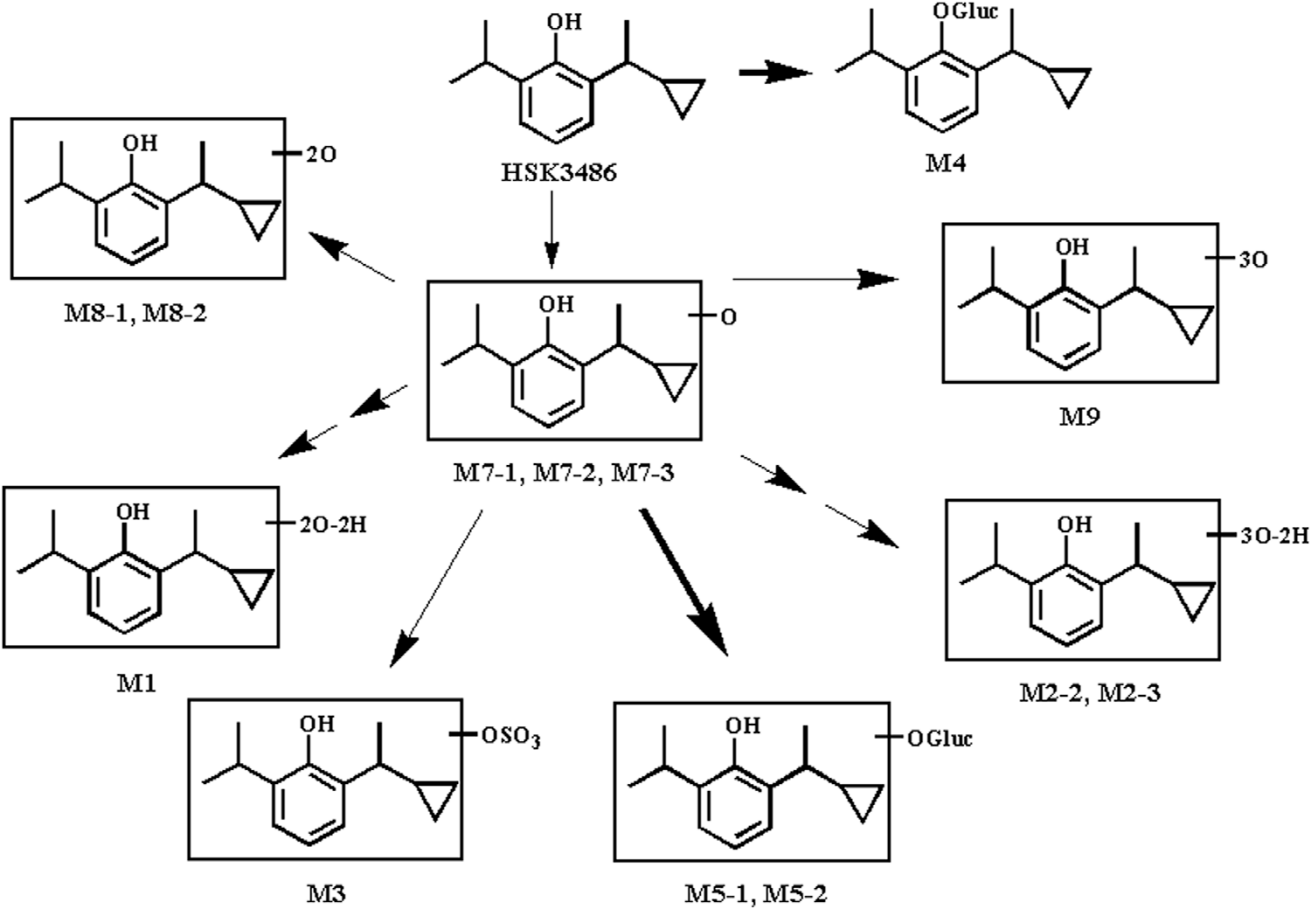
Proposed pathway of HSK3486 metabolism in microsomes or S9 fraction.

Although the major human oxidative metabolite M7-2 was not found in monkey, dog, or mouse liver microsomes, its glucuronide conjugate M5-1 was generated in liver S9 fractions from these species, indicating that M7-2 was also formed in animal liver. It is possible that the M7-2 metabolite in animal liver microsomes was further oxidized to M8 and M9. In liver S9 fractions, M7-2 reacted rapidly with glucuronic acid to form M5-1 and M5-2 under the catalysis of UDP-glucuronosyltransferases. Thus, hydroxylation and glucuronide conjugation are the major pathways of HSK3486 metabolism in the liver of these five species (Figure 7).

### Identification of Cytochrome P450 Enzymes Responsible for Metabolism of HSK3486

Significant metabolism of HSK3486 was observed in incubations with all heterologous expressed CYP enzymes tested: CYP1A2, CYP2A6, CYP2B6, CYP2C8, CYP2C9, CYP2C19, CYP2D6, CYP2E1, CYP3A4 and CYP3A5. The fast clearance of HSK3486 was observed with isoforms CYP1A2 (CLint = 6.77 μL/min/pmol), CYP2B6 (CLint = 65.1 μL/min/pmol) and CYP2C19(CLint = 7.87 μL/min/pmol) (Supplementary Table S7).

In human liver microsome incubations, the metabolism of HSK3486 was inhibited significantly in the presence of specific inhibitors of CYP1A2, CYP 2B6 or CYP2C19, when metabolism was inhibited, respectively, by 33.7, 94.9 and 38.0% (Supplementary Table S8). These results suggest that CYP2B6 is the major isoform involved in HSK3486 metabolism, with CYP1A2 and CYP2C19 contributing to a smaller extent.

## Discussion

Propofol, the most commonly used anesthesic, acts effectively *via* GABA_A_ receptors but frequently causes injection pain and has other limitations (Doenicke et al., 1996; Robinson et al., 1997; Diedrich and Brown, 2011). In this work, we characterized HSK3486, a 2,6-disubstituted alkylphenol that acts as a sedative hypnotic agent. Since HSK3486 has a higher liposolubility than propofol, the concentration of free molecule in the emulsion is significantly lower than that of propofol, so it may cause less injection pain. Here we found that HSK3486 acts against the α_1_β_2_γ_2_ subtype of GABA_A_ receptors and inhibits a wide range of CYP450 isozymes in mammalian species. Its pharmacokinetics and distribution indicate rapid metabolism and low accumulation after continuous infusion. Together, these findings argue for the drug’s strong potential as an alternative to propofol in the clinic.

To explore the mechanism of action of HSK3486, we tested the binding selectivity and function of HSK3486 on the α_1_β_2_γ_2_ subtype of GABA_A_ receptors. This GABA_A_ subtype is considered the most abundant, and it contributes to chloride current-mediated inhibitory postsynaptic potentials. Whole-cell patch-clamp experiments indicated that HSK3486 potentiated GABA-evoked chloride currents in α_1_β_2_γ_2_-transfected HSK293 cells more potently than propofol. HSK3486 did not directly activate the receptor independently of GABA until its concentration was at least 20-fold higher than concentrations enhanced GABA-induced activation. In radiolabeling competitive binding assays, we found that HSK3486 competed with TBPS and TBOB, but not muscimol, flunitrazepam, or Ro-15-178. This suggests that HSK3486 has weak or no affinity for benzodiazepine and GABA sites, but has high affinity towards the picrotoxin-binding site or has a allosteric inhibition of TBPS/TBOB on GABA_A_ receptors.

HSK3486 produced similar hypnosis to propofol in mice, rats, and dogs following bolus IV injection, with rapid onset and rapid recovery. The therapeutic index of HSK3486 was about 1.5 times that of propofol. During anesthesia, the effect of HSK3486 on the cardiovascular system was acceptable. In telemetry experiments, 4 mg/kg HSK3486 reduced MAP in rats by 20% while 16 mg/kg propofol reduced it by 30%, and the two treatments triggered LORR for similar duration.

The hypothalamic–pituitary–adrenal (HPA) axis is essential for human adaptation to stress. The use of anesthetics may interfere with the activity of HPA axis activity, particularly continuous administration of etomidate and benzodiazepines. Few data exist on the effect of propofol on adrenal function in humans (Besnier et al., 2017). Studies on the effect of propofol on cortex functionl are mainly *in vitro*. HSK3486 is a close relative of propofol, therefore, we speculate that the effect of HSK3486 on cortical function is minimal. But in tissue distribution study, we found that HSK3486 accumulated in the adrenals of rats. This may remind us that in following pre-clinical and clinical studies, we should focus on immediate and post-operative period effect of HSK3486 on adrenal cortex functionon in following pre-clinical and clinical studies to ensure the safety of continuous infusion of HSK3486.

Pharmacokinetic studies in rats and dogs showed that HSK3486 had good pharmacokinetic properties as a sedative/hypnotic agent. HSK3486 was efficiently cleared in rats and dogs. The drug was cleared from plasma much faster than from the liver, the tissue distribution was wide, and the Vss was much higher than the total volume of body fluid. In rats and beagle dogs, the plasma elimination half-life of HSK3486 was short, so no drug accumulation was observed.

Although our experiments suggest that HSK3486 exerts little effect on cardiovascular and respiratory systems, its full range of potential actions needs to be further explored. A phase III clinical trial of HSK3486 is underway for the induction of general anesthesia in elective surgery and in fiberoptic bronchoscopy (NCT 04111159). If the drug shows properties as promising in patients as here in animals, it may move closer to becoming a new option for patients and anesthesiologists.

## Data Availability

The original contributions presented in the study are included in the article/Supplementary Material, further inquiries can be directed to the corresponding authors.
