# Supplementary material for: Pharmacodynamics and Pharmacokinetics of HSK3486, a Novel 2,6-Disubstituted Phenol Derivative as a General Anesthetic
Source: Front Pharmacol. 2022 Feb 3;13:830791. doi: 10.3389/fphar.2022.830791 (PMC8851058; doi:10.3389/fphar.2022.830791)
Supplement: Supplementary file 1 [file Table1.DOCX]

**Table 1.** Affinity of HSK3486 towards CYP450, 2C19，GABA_A_ and NET

| Tests | Inhibition（%） |
| --- | --- |
| CYP450，2C19 | 50 |
| GABA_A_, Chloride Channel, TBPS | 101 |
| GABAA, Flunitrazepam, Central | -28 |
| GABAA, Muscimol, Central | -1 |
| GABAA, Ro-15-1788, Cerebellum | -6 |
| GABAA, Ro-15-1788, Hippocampus | 12 |
| Transporter, Norepinephrine (NET) | 51 |

**Table 2.** HD50, LD50 and TI of HSK3486 and propofol in rats

|  | HD_50_ (mg/kg) | LD_50_ (mg/kg) | TI |
| --- | --- | --- | --- |
| HSK3486 | 0.88 | 8 | 9.1 |
| propofol | 5.05 | 31.31 | 6.2 |

**Table 3.** Sedative Hypnotic Effect Duration following HSK3486 Bolus in rats(mean±SD, n=10)

| HSK3486 (Injectable Emulsion) | | | | | | | |
| --- | --- | --- | --- | --- | --- | --- | --- |
| Dose of LORR(mg/kg) | 1 | 1.5 | 2 | 3.5 | 5 | 7.5 | 10 |
| Onset of LORR(s) | No LORR | 15-30 | ＜15 | ＜15 | ＜15 | ＜15 | ＜15 |
| LORR duration (min) | ﹣ | 5.7±3.9 | 5.9±3.0 | 14.8±3.3 | 16.1±5.8 | 26.4±7.3 | 36.4±3.2 |
| Recovery time (min) | ﹣ | 1.1±0.9 | 3.0±2.0 | 0.9±1.4 | 4.1±5.2 | 1.3±2.1 | 3.0±1.7 |

**Table 4.** Pharmacokinetic Parameters for HSK3486 after a Single Injection in Male and Female Rats

| Dose (mg/kg) | N | AUC_0-t_（h*ng/mL) | AUC_0-∞_（h*ng/mL) | Cmax (ng/mL) | CL (L/h/kg) | t_1/2_ (h) | Vss (mL/kg) |
| --- | --- | --- | --- | --- | --- | --- | --- |
| 1 | 6 | 55.8 | 57.3 | 236 | 18.2 | 0.46 | 6.84 |
| 2 | 6 | 130 | 135 | 459 | 15.1 | 0.95 | 8.38 |
| 4 | 6 | 286 | 295 | 1046 | 13.8 | 0.75 | 8.16 |

AUC = area under concentration-time curve; C_max_=maximum concentration observed;

t_1/2_= half-time; CL=clearance; Vss= volume of distribution at steady-state

**Table 5.** Sedative Hypnotic Effect of HSK3486 Bolus and Propofol in Beagle Dogs (mean±SD, n=6)

|  | HSK3486 (Injectable Emulsion) | | | Propofol | | |
| --- | --- | --- | --- | --- | --- | --- |
| Dose(mg/kg) | 0.8 | 1.2 | 2.5 | 3 | 5 | 10 |
| Onset time(min) | 1.33±0.42 | 1.04±0.26 | 0.77±0.35 | 1.49±0.35 | 1.04±0.38 | 0.64±0.13 |
| Anesthesia time (min) | 7.09±3.41 | 13.14±7.02 | 28.14±5.26 | 6.40±3.01 | 14.29±5.43 | 23.59±8.93 |
| Walking without ataxia (min) | 3.06±2.43 | 2.13±2.89 | 1.05±0.38 | 3.17±3.18 | 1.93±1.91 | 2.59±2.73 |

**Table 6. Pharmacokinetic Parameters of HSK3486 after a Single Dose Injection in Dogs**

| Dose (mg/kg) | N | AUC_0-t_（h*ng/mL) | AUC_0-∞_（h*ng/mL) | Cmax (ng/mL) | CL (L/h/kg) | t_1/2_ (h) | Vss (mL/kg) |
| --- | --- | --- | --- | --- | --- | --- | --- |
| 1 | 6 | 165 | 179 | 564 | 6.67 | 1.28 | 7.36 |
| 2 | 6 | 342 | 387 | 1214 | 5.32 | 1.66 | 5.75 |
| 4 | 6 | 716 | 790 | 2796 | 5.25 | 1.37 | 5.06 |

AUC, area under concentration-time curve; C_max_, maximum concentration observed;

t_1/2_, half-life; CL,clearance; Vss,volume of distribution at steady-state

**Table 7. The metabolism parameter of HSK3486 in human liver microsomes with and without CYP isoforms chemical inhibitors (n=3)**

| CYP Isoform (Inhibitors) | Remaining Percentage (%) at 5 min | Remaining Percentage (%) at 60 min | Inhibition Percentage (%) |
| --- | --- | --- | --- |
| Without Inhibitor | 16.7±1.76 | <1% | 0.00 |
| CYP1A2 (Furafylline) | 42.4 ±5.37 | <1% | 33.7 |
| CYP2B6 (Ticlopidine) | 91.3 ±1.37 | 47.9 ± 4.31 | 94.9 |
| CYP2C8 (Montelukast) | 15.3 ±0.91 | <1% | -49.8 |
| CYP2C9 (Sulfaphenazole) | 14.8 ±2.20 | <1% | -52.8 |
| CYP2C19 (N-3-benzylnirvanol) | 32.9 ±2.20 | <1% | 38.0 |
| CYP2D6 (Quinidine) | 25.3 ±6.92 | <1% | -12.9 |
| CYP3A4 (Ketoconazole) | 24.6 ±1.33 | <1% | -11.9 |

**Table 8. The Intrinsic clearance (Cl_int_) and half-life (t_1/2_) of HSK3486 incubated with individual recombinant human CYP isoforms (mean ± SD, n=3)**

| **CYP Isoform** | **Cl_int_ (µL/min/pmol CYP)** | **t_1/2_ (min)** |
| --- | --- | --- |
| CYP1A2 | 6.77±0.527 | 2.06±0.154 |
| CYP2A6 | 0.405±0.00818 | 34.2±0.698 |
| CYP2B6 | 65.1±0.739 | 0.213±0.00242 |
| CYP2C8 | 0.407±0.0137 | 34.1±1.14 |
| CYP2C9 | 0.710±0.0561 | 19.6±1.51 |
| CYP2C19 | 7.87±0.287 | 1.76±0.0631 |
| CYP2D6 | 0.486±0.0579 | 28.8±3.57 |
| CYP2E1 | 0.360±0.000702 | 38.5±0.0752 |
| CYP3A4 | 1.78±0.420 | 8.10±2.14 |
| CYP3A5 | 0.455±0.0482 | 30.7±3.45 |
